# Successful embolization of subcutaneous mesenteric varices within an ileal conduit in a patient with liver cirrhosis

**DOI:** 10.1002/iju5.12644

**Published:** 2023-09-20

**Authors:** Ayaka Sakurai, Akira Ohtsu, Seiji Arai, Masanori Aoki, Miho Ikeya, Hiroyuki Tokue, Keisuke Hori, Yuji Fujizuka, Yoshitaka Sekine, Hidekazu Koike, Kazuhiro Suzuki

**Affiliations:** ^1^ Department of Urology Gunma University Hospital Maebashi Gunma Japan; ^2^ Department of Diagnostic and Interventional Radiology Gunma University Hospital Maebashi Gunma Japan

**Keywords:** embolization, ileal conduit, liver cirrhosis, mesenteric veins, varices

## Abstract

**Introduction:**

Venous hemorrhage from ectopic varices is potentially fatal. This report describes a rare case in which bleeding from mesenteric varices in an ileal conduit was treated successfully by embolization therapy.

**Case presentation:**

The patient was an 82‐year‐old man who had previously undergone total pelvic exenteration for colon cancer with creation of an ileal conduit for urinary diversion. He subsequently developed liver cirrhosis and underwent partial hepatectomy for hepatocellular carcinoma. 9 years after his colon surgery, he was admitted with gross hematuria. Computed tomography revealed subcutaneous mesenteric varices in the ileal conduit and hemorrhage as a result of rupture of the varices. The bleeding continued despite repeated manual compression but was eventually stopped by embolization therapy.

**Conclusion:**

Embolization therapy may be helpful for hemostasis in the event of intractable bleeding from mesenteric varices in an ileal conduit.

**Abbreviations & Acronyms**
BRTO: balloon‐occluded retrograde transvenous obliteration
CT: computed tomography
TIPS: transjugular intrahepatic portosystemic shunt

**Keynote message**

Portal hypertension induced ectopic mesenteric varices within an ileal conduit in a patient with liver cirrhosis. Despite potentially life‐threatening bleeding from the varices, the patient was treated successfully by embolization therapy. Embolization may be a suitable treatment for intractable bleeding from mesenteric varices in an ileal conduit.

## Introduction

Most ectopic varices occur in patients with portal hypertension and liver cirrhosis and often lead to hemorrhage, which is potentially fatal. In this report, we describe a rare case in which we used embolization therapy for repeated bleeding from subcutaneous mesenteric varices in an ileal conduit in a patient with liver cirrhosis.

## Case presentation

The patient was an 82‐year‐old man who had previously undergone total pelvic exenteration for colon cancer with bladder invasion (pT4bN1M0) with the construction of an ileal conduit. He subsequently received chemotherapy and radiotherapy for lymph node metastases. 6 years after the surgery, he developed bilateral hydronephrosis, which was treated with a single J ureteral stent in a retrograde manner via the ileal conduit. 7 years after the colon surgery, he underwent partial hepatectomy for hepatocellular carcinoma. He also had alcoholic liver cirrhosis and was taking an anticoagulant after coronary artery bypass surgery for angina.

**Fig. 1:**
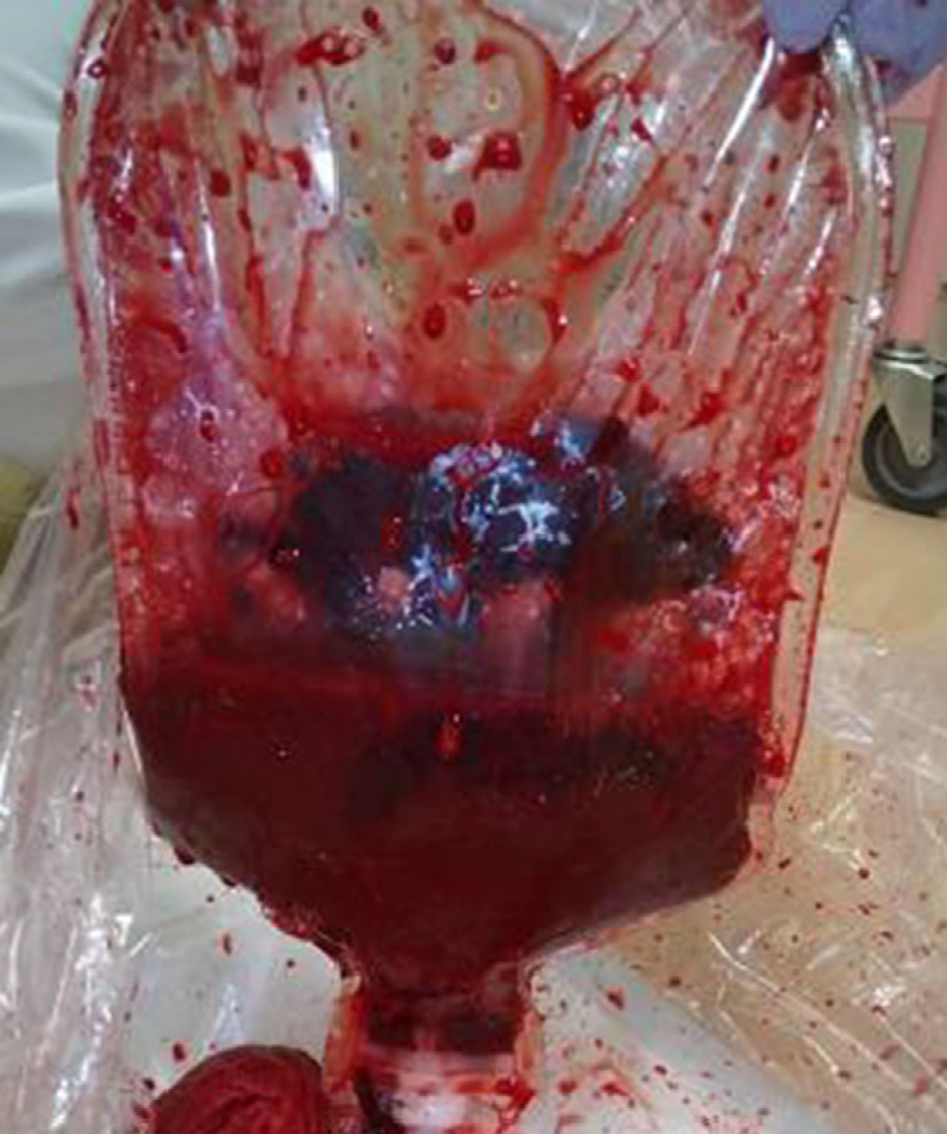
Image of the stoma pouch. The pouch contained 500 mL of gross hematuria with blood clots. The rate of blood loss was approximately 300 mL/h.

**Fig. 2:**
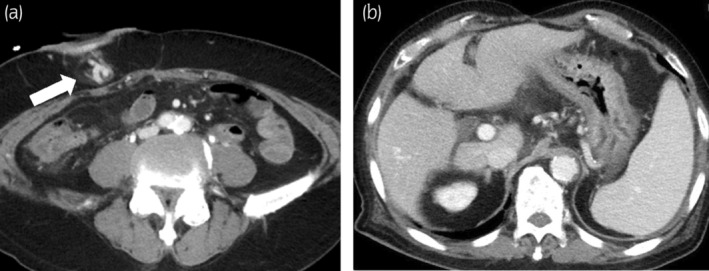
CT images of the mesenteric varices and liver cirrhosis. Images were acquired at the time of hemorrhage (a). The white arrow indicates expansion of the mesenteric vein. CT also revealed liver cirrhosis and splenomegaly (b).

**Fig. 3:**
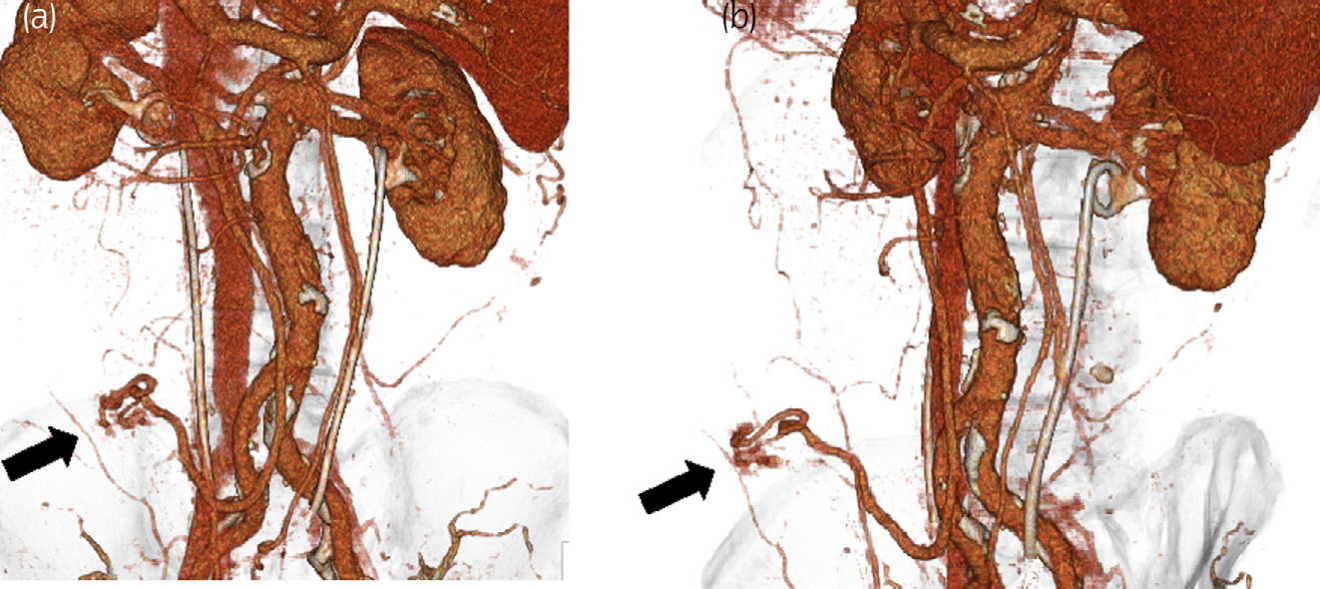
Three‐dimensional constructions of the mesenteric varices in the ileal conduit before embolization therapy. The black arrows indicate expansion of the mesenteric vein (a,b).

Nine years after the colon surgery, the patient was admitted with blood clots in the stoma pouch (Fig. 1). Renal pelvic lavage with single J stents revealed no gross hematuria from the kidneys, and flexible cystoscopy did not show bleeding inside the ileal conduit. Instead, the hemorrhage was tracked to the outside wall of his ileal conduit. Laboratory investigations revealed slight anemia, thrombocytopenia, and a prolonged prothrombin time. The Child‐Pugh score was class B. Contrast‐enhanced CT revealed stable liver cirrhosis and expansion of the subcutaneous mesenteric veins (Figs 2, 3). Black stools were noted at this time, and gastrointestinal endoscopy revealed esophageal varices at risk of imminent rupture. The initial plan was to ligate the esophageal varices endoscopically. However, even with cessation of the anticoagulant and manual compression, repeated episodes of bleeding from the outside wall of the ileal conduit continued, and transfusions for progressive anemia could not be avoided. Therefore, we opted to treat the subcutaneous mesenteric varices by interventional radiology despite the risk of worsening esophageal varices.

**Fig. 4:**
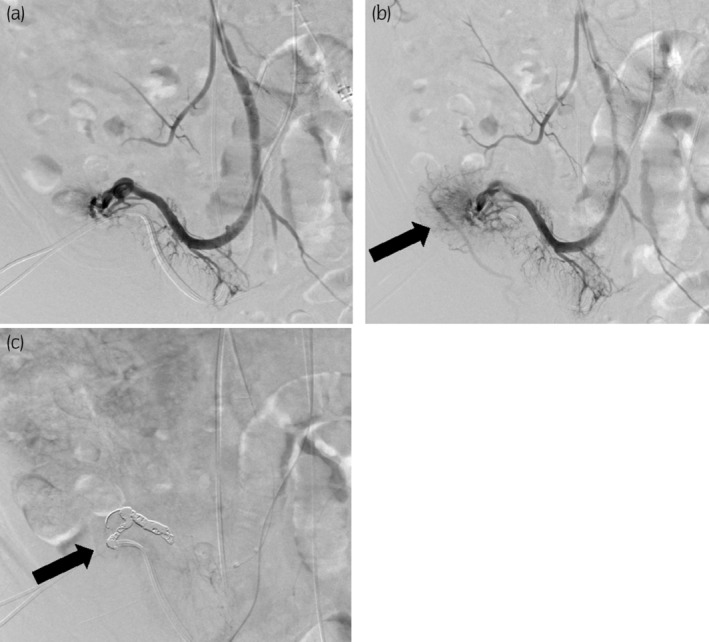
Images of embolization therapy. Ileal venography revealed ectopic mesenteric varices (a). Ileal venography under balloon occlusion identified the varices in the ileal conduit and the right inferior epigastric vein (b). The black arrow indicates mesenteric varices and the outflow tract (right inferior epigastric vein). Metal coil embolization therapy was implemented for the mesenteric varices (c). The black arrow indicates the micro‐coils.

Selective superior mesenteric angiography did not reveal arterial bleeding but detected well‐developed ileal conduit varices. We found that the outflow tract of the varices connected with the right inferior epigastric vein. We then treated the subcutaneous mesenteric varices by BRTO. Under ultrasound guidance, we punctured the left branch of the portal vein and performed portal angiography. A 5‐Fr balloon catheter was inserted into the portal vein to reach the ileal vein (Fig. 4a,b). With balloon occlusion of the ileal vein and manual compression of the right inferior epigastric vein, dextrose solution, and monoethanolamine oleate were administered to the varices and allowed to pool for 20 minutes. The varices were then embolized with micro‐coils (Fig. 4c). Finally, angiography via the superior mesenteric artery confirmed disappearance of the varices. After BRTO, we found slightly more red‐colored esophageal varices than before and ligated them endoscopically. Thereafter, there was no further bleeding from the mesenteric or esophageal varices, and the patient was discharged from hospital.

## Discussion

Bleeding ectopic varices as a result of portal hypertension is a rare cause of hematuria in patients with urinary diversion using an intestinal segment.^1^ When hemorrhage occurs, it can become a severe problem and has a mortality rate of 3%.^2^, ^3^ Importantly, a previous study found that ileal varices were present in 18% of patients with liver cirrhosis.^4^ The circulating venous network is isolated by ileal segmentation, potentially resulting in increased venous pressure in an ileal conduit.

Diagnosis of ileal conduit varices is complicated and requires detailed examination of the stoma site. The location of the varices and the clinical symptoms determine the treatment method while considering the risks and benefits.^5^ Interventional radiology is a minimally invasive image‐guided treatment, and for portal hypertension is divided into variceal embolization and portal pressure reduction.^6^ Variceal embolization includes BRTO, percutaneous transhepatic obliteration, and direct percutaneous embolization, while portal pressure reduction consists of TIPS.^3^ To date, there have been no studies comparing the efficacy of embolization with that of TIPS in patients with ileal conduit varices. Conversely, embolization with BRTO is a more effective treatment than TIPS for gastric varices.^7^, ^8^ Importantly, BRTO does not reduce portal flow and can avoid further worsening of hepatic function.^9^ Therefore, we selected BRTO for safe treatment of the ileal conduit varices in this patient. Direct percutaneous embolization could be an alternative method because we could see the varices with ultrasound (Fig. S1). A potential risk with BRTO is worsening of elevated portal pressure.^10^ Indeed, in this patient, esophageal endoscopy after BRTO revealed slightly more red‐colored esophageal varices than before. If a patient has esophageal varices at risk of imminent rupture, endoscopic ligation of esophageal varices should be performed before or immediately after BRTO of mesenteric varices. Another risk with BRTO is re‐bleeding after embolization.^10^ Fortunately, this did not occur in our patient; however, he remains under close follow‐up.

Depending on the severity of the underlying liver cirrhosis, development of ectopic mesenteric varices after urinary diversion surgery has been estimated to take an average of 19 months to 24 years.^4^ Although contrast‐enhanced CT images were repeatedly obtained after surgery, expansion of the mesenteric veins was not detectable in this patient. 2 years before the present admission, he had undergone partial hepatectomy for hepatocellular carcinoma with alcoholic liver cirrhosis. It may be that his portal vein pressure increased slowly with the liver cirrhosis and subsequent partial hepatectomy, culminating in bleeding from mesenteric varices 9 years after the surgery. Ureterostomy does not entail use of an intestinal segment, and there have been no reports of bleeding from ectopic varices in the ureter after ureterostomy for portal hypertension. Ureterostomy could be a better choice for urinary diversion at the time of colon surgery in a patient with liver cirrhosis.^11^

In this case, portal hypertension induced ectopic mesenteric varices in the ileal conduit, and the intractable bleeding from the varices worsened with thrombocytopenia and anticoagulant therapy. This case indicates that embolization with BRTO may be a suitable hemostasis method for repeated hemorrhage of mesenteric varices from an ileal conduit.

## Author contributions

Ayaka Sakurai: Conceptualization; data curation; investigation; visualization; writing – original draft. Akira Ohtsu: Conceptualization; data curation; investigation; visualization; writing – original draft. Seiji Arai: Conceptualization; data curation; investigation; project administration; supervision; visualization; writing – original draft; writing – review and editing. Masanori Aoki: Data curation. Miho Ikeya: Visualization; writing – original draft; writing – review and editing. Hiroyuki Tokue: Visualization; writing – original draft; writing – review and editing. Keisuke Hori: Data curation. Yuji Fujizuka: Data curation. Yoshitaka Sekine: Data curation; writing – review and editing. Hidekazu Koike: Data curation. Kazuhiro Suzuki: Data curation; writing – review and editing.

## Conflict of interest

The authors declare that they have no competing interests.

## Informed consent

Written informed consent for publishing this case report was obtained from the patient.

## Registry and the Registration No. of the study/trial

Not applicable.

## Approval of the research protocol by an Institutional Review Board

Not applicable.

## Funding information

The authors have no funding to declare for this article.

## Supporting information


**Figure S1** Ultrasonographic image showing subcutaneous mesenteric varices (white arrow).
