# Supplementary material for: Successful embolization of subcutaneous mesenteric varices within an ileal conduit in a patient with liver cirrhosis
Source: IJU Case Rep. 2023 Sep 20;6(6):445–8. doi: 10.1002/iju5.12644 (PMC10622204; doi:10.1002/iju5.12644)
Supplement: Supplementary file 1 — Figure S1 Ultrasonographic image showing subcutaneous mesenteric varices (white arrow). [file IJU5-6-445-s001.pptx]

## Slide 1
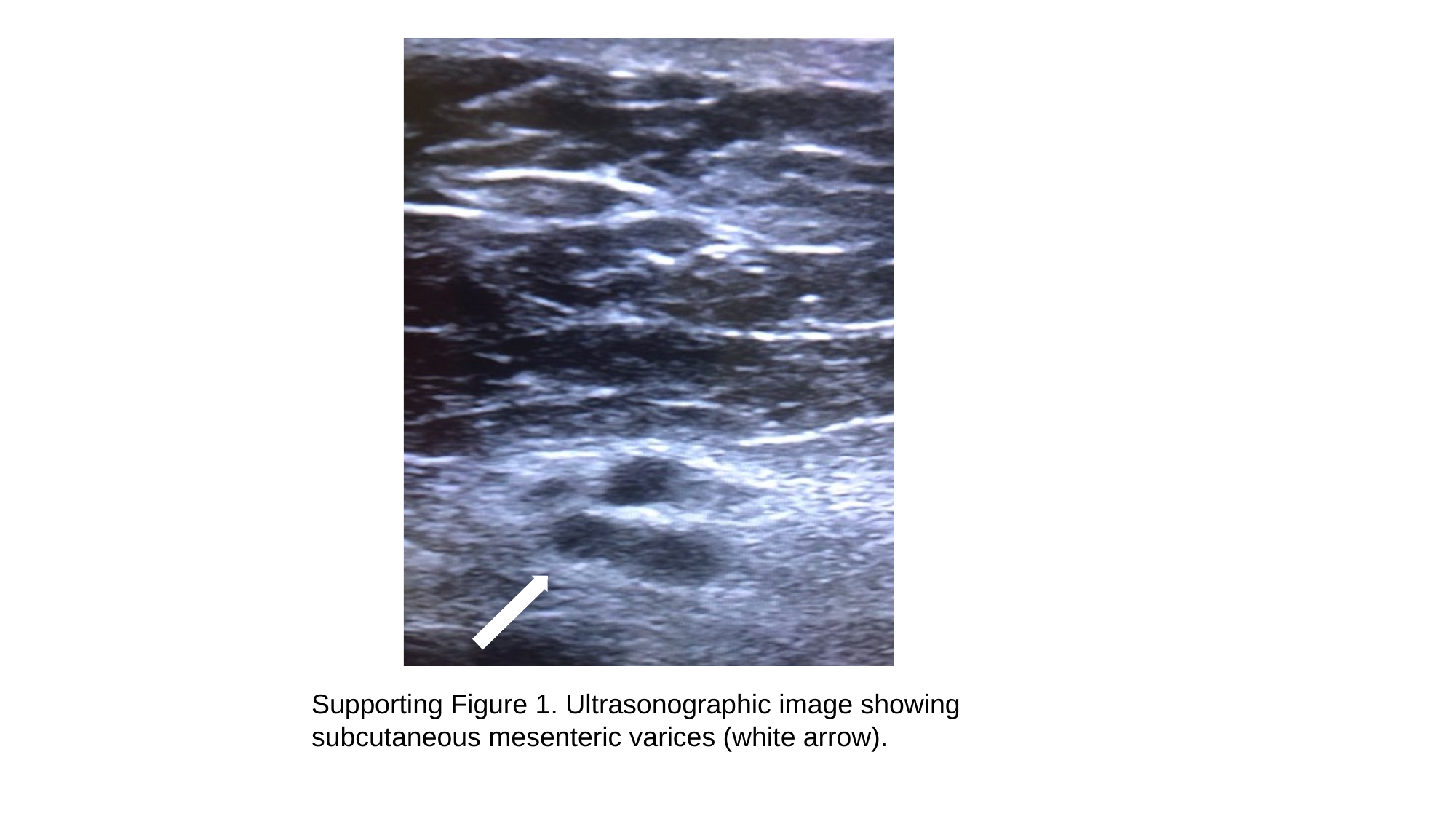

Supporting Figure 1. Ultrasonographic image showing
subcutaneous mesenteric varices (white arrow).
